# Hybrid Multilevel Sparse Reconstruction for a Whole Domain Bioluminescence Tomography Using Adaptive Finite Element

**DOI:** 10.1155/2013/548491

**Published:** 2013-03-03

**Authors:** Jingjing Yu, Xiaowei He, Guohua Geng, Fang Liu, L. C. Jiao

**Affiliations:** ^1^School of Physics and Information Technology, Shaanxi Normal University, Xi'an, Shanxi 710062, China; ^2^School of Information Sciences and Technology, Northwest University, Xi'an, Shanxi 710069, China; ^3^School of Computer Science and Technology, Xidian University, Xi'an, Shanxi 710071, China; ^4^Key Laboratory of Intelligent Perception and Image Understanding of Ministry of Education of China, Xi'an, Shanxi 710071, China

## Abstract

Quantitative reconstruction of bioluminescent sources from boundary measurements is a challenging ill-posed inverse problem owing to the high degree of absorption and scattering of light through tissue. We present a hybrid multilevel reconstruction scheme by combining the ability of sparse regularization with the advantage of adaptive finite element method. In view of the characteristics of different discretization levels, two different inversion algorithms are employed on the initial coarse mesh and the succeeding ones to strike a balance between stability and efficiency. Numerical experiment results with a digital mouse model demonstrate that the proposed scheme can accurately localize and quantify source distribution while maintaining reconstruction stability and computational economy. The effectiveness of this hybrid reconstruction scheme is further confirmed with *in vivo* experiments.

## 1. Introduction

Bioluminescence imaging (BLI) is an *in vivo* imaging modality that has been successfully used in preclinical researches [1–3]. This imaging strategy exploits the properties of luciferase that can generate visible or near infrared light through the oxidation of an enzyme-specific substrate in the presence of oxygen and adenosine triphosphate [4]. As the produced light intensity is directly proportional to the concentration of luciferase-expressing cells, BLI can reveal cellular and molecular features of biology and disease [5]. However, BLI fails to provide depth information of the internal biological sources [6]. Collecting measurement data from multiple views or combining multiple BLI acquisition with geometrical structures acquired by micro-CT or MRI, bioluminescence tomography (BLT) tries to reconstruct the 3D biological source distribution. In this way, BLT overcomes the limitation of planar imaging in poor spatial resolution and further facilitates our understanding of biomolecular processes as they occur in living animals. Therefore, BLT has substantial potential to be a powerful tool for noninvasively monitoring and tracking a variety of biological processes [7].

Generally, BLT involves a forward and an inverse problem (source reconstruction). Due to the diffusive nature of photon propagation in tissue, BLT source reconstruction is known to be a highly ill-posed problem [6, 8]. To overcome the inherent ill-posedness of the tomographic problem in BLT, different strategies have been proposed either by increasing the amount of independent measurements with spectrally resolved or multispectral approaches [9–13] or by reducing the number of unknowns with permissible source region [6, 10, 13, 14]. Up to now, quantitative reconstruction for whole domain BLT with monochromatic boundary measurements has not been intensively investigated.

As in many other imaging modalities, the achievable resolution for BLT is determined firstly by the signal to noise ratio, and secondly by the level of discretization. Image quality can be improved by uniformly refining mesh throughout the reconstruction domain. Nevertheless, global refinement tends to further aggravate the ill-posedness and incur insurmountable computational burden due to the increased unknowns and problem size. Consequently, the use of adaptive finite element method (AFEM) is an indispensable approach to improve image quality [15–21].

In this contribution, we present a whole domain BLT method based on AFEM which provides fine resolution around targets with coarser resolution in other regions. Unlike the previous AFEM-based BLT that adopted identical inversion strategy on different mesh levels [15, 18–21], we take the variance on different discretization levels into account and propose a novel hybrid multilevel reconstruction scheme to maintain solution stability and computational economy. Two different inversion algorithms, the stagewise fast LASSO (SwF-LASSO) [22] and the incomplete variables truncated conjugate gradient method (IVTCG) [23], are applied to the first mesh level and the succeeding ones according to their respective characteristics.

The following sections describe some of the implementation details of the hybrid AFEM algorithm, the evaluations on a digital mouse model, and the validation with an *in vivo* experiment. Short discussions and concluding remarks are given at the end of this paper.

## 2. Methodology

### 2.1. Photon Propagation Model

In this work, we assume that the structural and optical parameters regarding different organs are given. Therefore, the BLT reconstruction comes down to a linear inverse source problem. Based on the diffusion approximation model of radiative transfer equation, a linear relationship between the source distribution and boundary measurements is then derived with the finite element method [6]:

(1) $$\begin{array}{c} AS=\Phi^{m}, \end{array}$$

where *A* ∈ *R* ^*M*×*N*^ (*M* < *N*) is the system matrix, *S* ∈ *R* ^*N*^ denotes the internal source distribution, and Φ^*m*^ ∈ *R* ^*M*^ represents measurable boundary nodal photon density that is usually calculated from the surface flux image captured by a CCD camera.

In view of the limitation of using permissible source region in BLT reconstruction, we consider a whole domain reconstruction scheme without this kind of* a priori* information. On the other hand, *l*_1_-norm based sparse regularization methods have attracted considerable amount of attention in BLT [10, 20–25], and the reconstructions' results therein demonstrate that *l*_1_-norm solution fits the sparsity nature of bioluminescent source distribution in BLT practice. Using *l*_1_ regularization, we formulate the BLT inverse problem to the following optimization problem:

(2) $$\begin{array}{c} \underset{S}{\min}\frac{1}{2}{||AS-\Phi^{m}||}_{2}^{2}+\tau {||S||}_{1}, \end{array}$$

where ||·||_2_ denotes the Euclidean norm, ||·||_1_ is the *l*_1_ norm, and *τ* > 0 is a regularization parameter.

### 2.2. Hybrid Multilevel Reconstruction Based on AFEM

In order to provide the resolution necessary for imaging at acceptable computational cost, the domain *Ω* is dynamically discretized into a nested sequence of tetrahedral meshes {Θ_1_,…Θ_*k*_,…}, rather than a fixed and uniformly fine mesh. In the proposed hybrid multilevel AFEM reconstruction process, reconstruction starts at the coarsest level and proceeds to the finer ones by locally refining the particular region based on a previous reconstruction procedure.

We note that the first reconstructed procedure on the coarsest mesh is quite different from the subsequent ones in the following three aspects. (i) It is based on a uniform mesh while others are with a locally refined mesh. (ii) The inversion on the first discretization level involves a large-size underdetermined system. In contrast, all of the subsequent reconstructions on locally finer region involve overdetermined systems. (iii) It has no *a priori* information of a promising region in whole domain case, whereas the others can obtain a permissible source region to constrain the solution space from a previous reconstruction procedure. Consequently, the specific inversion should be different on different meshes, and thus we propose a hybrid multilevel reconstruction scheme.

On the first mesh Θ_1_, we employed the recently reported greedy algorithm SwF-LASSO to solve the underdetermined problem in (2). The SwF-LASSO algorithm converges very fast and is able to find an approximate value close to the real distribution in only a few iteration steps. A brief outline of SwF-LASSO is given as follows [22].

**Step 0**

Initialization. *n* = 0, index set *O* = {1,2,…*N*}, *P* = Φ.

**Step 1**

Selecting basis function.

For *i* ∈ *O*, compute Δ*L*_*i*_^*n*+1^ = −(*q*_*i*_^*n*^)^2^/*a*_*i*_^*T*^ *a*_*i*_, compute the stagewise threshold $\gamma =\sqrt{\sum_{i\in O}{\left(\Delta L_{i}^{n+1}\right)}^{2}/\left|O\right|}$ and then determine the index set of the basis functions to be selected *K* ^*n*+1^ = {*i* : Δ_0_ > |Δ*L*_*i*_^*n*+1^| > *c* · *γ*,  *i* ∈ *O*}.

**Step 2**

The algorithm will be terminated when the index set *O* = Φ is empty, or |max⁡_*i*∈*K*^*n*+1^_Δ*L*_*i*_^*n*+1^| < *ε*, or *K* ^*n*+1^ = Φ.

**Step 3**

Update variables.

(3) $$\begin{array}{c} Q^{n+1}=\left(\begin{array}{cc} Q^{n} & 0\\ 0^{T} & 0 \end{array}\right)+\eta \left(\begin{array}{c} \rho \\ -1 \end{array}\right)\left(\begin{array}{cc} \rho^{T} & -1 \end{array}\right), \end{array}$$

where *ρ* = *Q* ^*n*^ *A*_*P*_^*T*^ *A*_*K*_, *η* = (*A*_*K*_^*T*^ *A*_*P*_ − *A*_*K*_^*T*^ *A*_*P*_ *ρ*)^−1^ and *A*_*K*_ consists of those column vectors of *A* relating to the selected basis functions in *K* ^*n*+1^. The updating formula of *S* is

(4) $$\begin{array}{c} \left(\begin{array}{c} S_{P}^{n+1}\\ S_{K}^{n+1} \end{array}\right)=\left(\begin{array}{c} S_{P}^{n}\\ 0 \end{array}\right)+\left(\begin{array}{c} \rho \eta \Delta \\ -\eta \Delta \end{array}\right), \end{array}$$

where Δ = *ρ* ^*T*^(*A*_*P*_^*T*^Φ^*m*^ − *λν*_*P*_/2) − *A*_*K*_^*T*^Φ^*m*^ + *λν*_*K*_/2.

**Step 4**

*O* = *O* − *K* ^*n*+1^, and *P* = *P* + *K* ^*n*+1^.

**Step 5**

*n* = *n* + 1, go to Step 1.

After the inversion on Θ_*i*_ (*i* = 1,2, 3,…) completes, adaptive mesh refinement is triggered. All of the elements with nonzero reconstructed value are selected to be refined, which can be regarded as a kind of mesh refinement strategy based on *posteriori* error estimation. Using the longest-edge bisection method, a locally refined mesh Θ_*i*+1_ is obtained [17].

Unlike other previous reports, we employ a different reconstruction procedure on the succeeding mesh levels Θ_*i*_ (*i* > 1). The IVTCG algorithm proposed in [23] has been demonstrated as an effective reconstruction method by reformulating (2) as a convex quadratic program with nonnegative constrained conditions. It updates only partial variables in working set per iteration and adopts a working set splitting strategy to find the searching direction more efficiently, which leads to a small subproblem to be minimized and greatly decreases the number of iterations. The model transformation and the mechanism of IVTCG are detailed in [23].

We note that it is the sparseness-related parameter *N*_*s*_ that controls the size of the subproblem, which is solved by the truncated conjugate gradient method. Generally, for a very sparse problem, IVTCG can obtain accurate results with reasonable computational efficiency by setting *N*_*s*_ = ⌊*M*/10⌋ and the maximum iterate number of the subproblem iter_max⁡_ = *N*_*s*_. However, in the reconstruction procedures after local mesh refinement, the target is not a very sparse signal and the computational cost will increase sharply. In view of this feature, we make a modification and adjust the parameter *N*_*s*_ = ⌊*M*/4⌋, and iter_max⁡_ = 25 in our implementation.

A new round of local mesh refinement and reconstruction will be performed until the number of refinement exceeds the maximum number *k*_max⁡_ or the model misfit ||*AS*−Φ^*m*^||_2_^2^ is reduced below a prespecified threshold *ε*. For the results reported in this work, we used *k*_max⁡_ = 4 and *ε* = 10^−5^.

**Figure 1:**
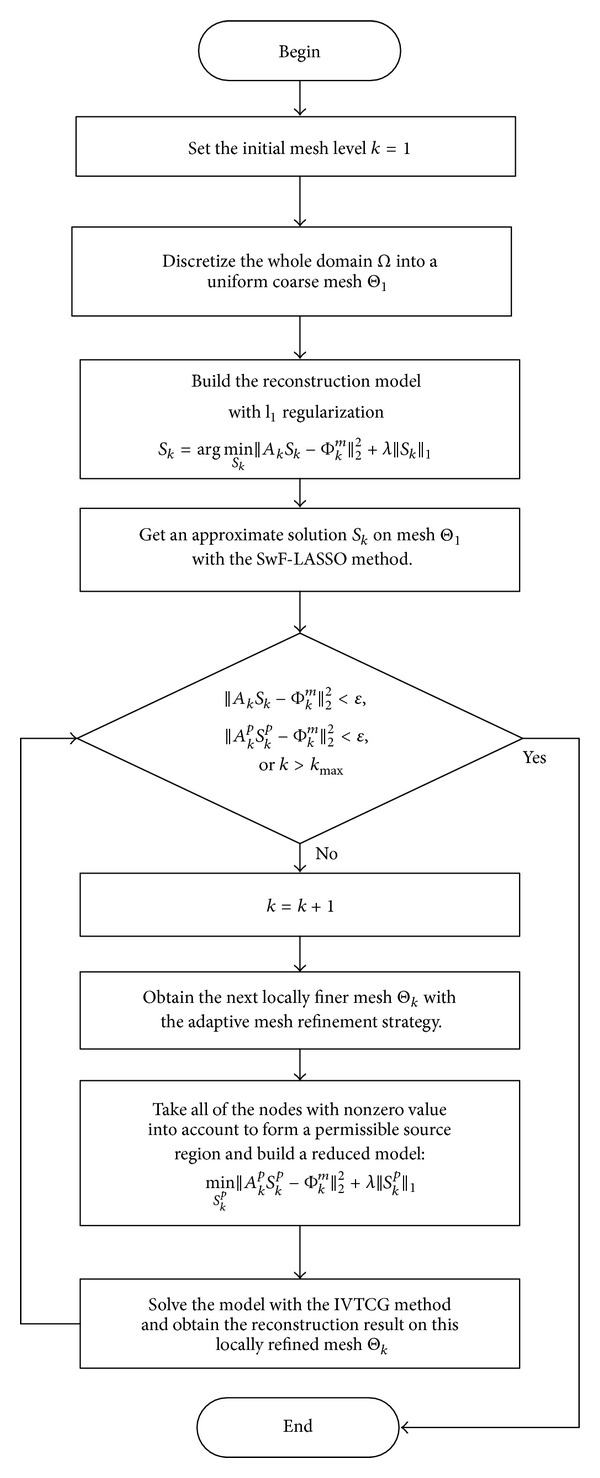
Flow chat of the hybrid multilevel reconstructions method.

The procedure of the proposed hybrid multilevel reconstructions method is illustrated in Figure 1.

## 3. Numerical Experiments and Results

We tested the proposed hybrid multilevel reconstruction method with a digital mouse model employing synthetically generated data. In the following simulations, we employed a 3D mouse atlas of CT and cryosection data to provide anatomical information [26]. The CT slices of the mouse were segmented into major anatomical components, including lungs, a heart, a liver, a stomach, kidneys, and muscles. The corresponding optical properties were the same as the settings in [27], as shown in Table 1. The whole region included the mouse torso with a height of 45 mm.

In the following numerical experiments, the torso model was discretized into a tetrahedral-element mesh, and synthetic measurements were generated by solving the forward model with FEM. To simulate the noise involved in real BLT experiments, 15% Gaussian white noise was added to the synthetic data. The qualities of the reconstruction are quantitatively assessed in terms of location error (LE) and relative error (RE) between the reconstructed power and the actual value.

### 3.1. Single-Target Reconstruction

**Figure 2:**
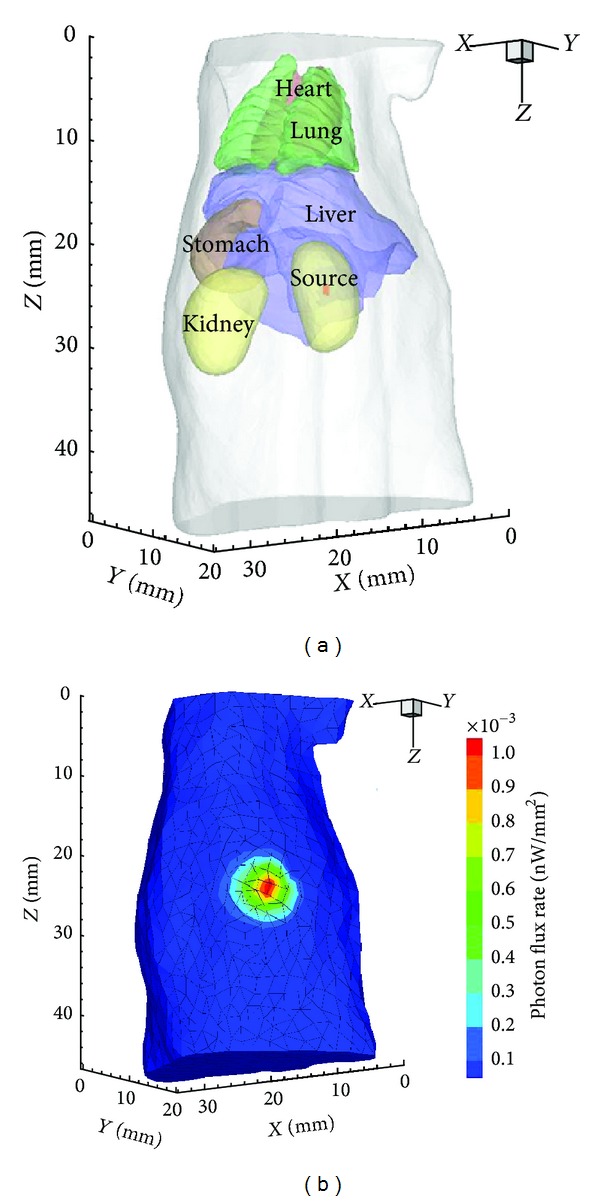
(a) The torso of the mouse atlas model with a cylindrical source in the right kidney. (b) Initial mesh for reconstruction and the simulated photon distribution on surface.

In the first set of experiments, a cylindrical source with 0.4 mm radius and 1 mm height was positioned in the right kidney with the center at (11, 6, 25), as shown in Figure 2(a). The actual source power was 0.2299 nW after discretization with FEM.  Figure 2(b) shows the initial mesh for reconstruction and the photon distribution on the surface.

**Figure 3:**
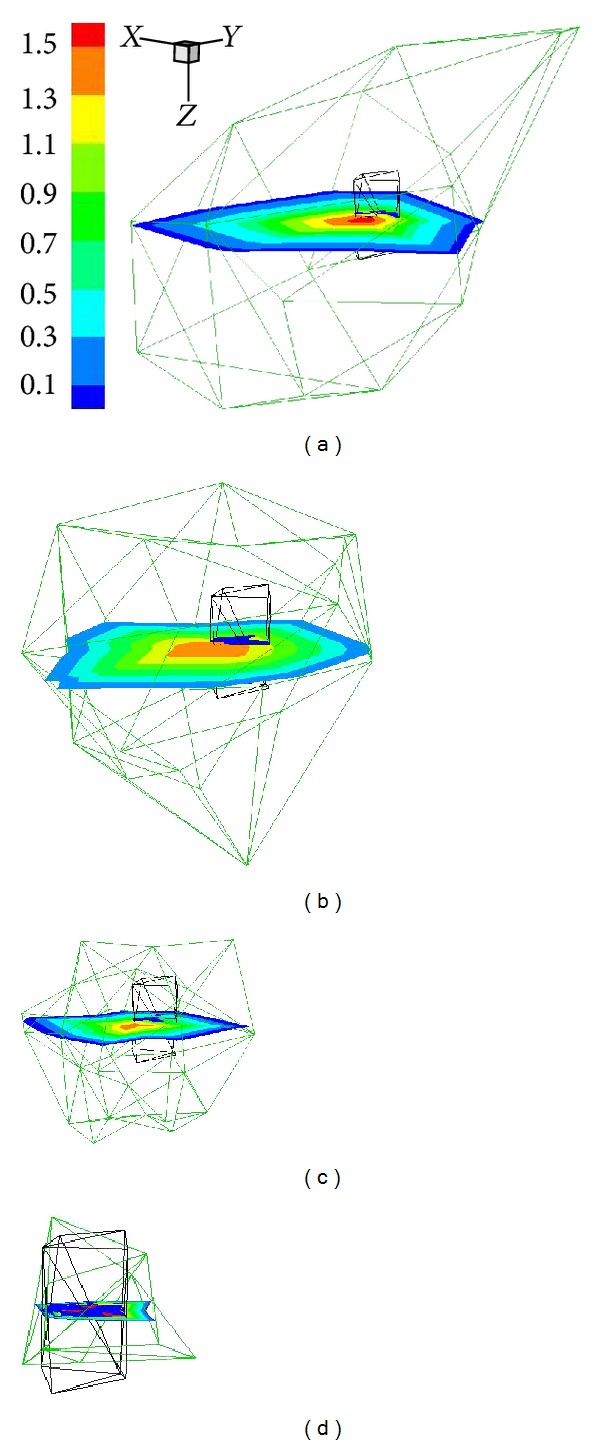
Evolution of reconstruction results with the local refined mesh in the single-source case. The green mesh denotes the local region that consists of nonzero nodes of the solution; the black mesh is the discretized source. (a) to (d) corresponds to four mesh levels, that is, Θ_1_ to Θ_4_.

**Figure 4:**
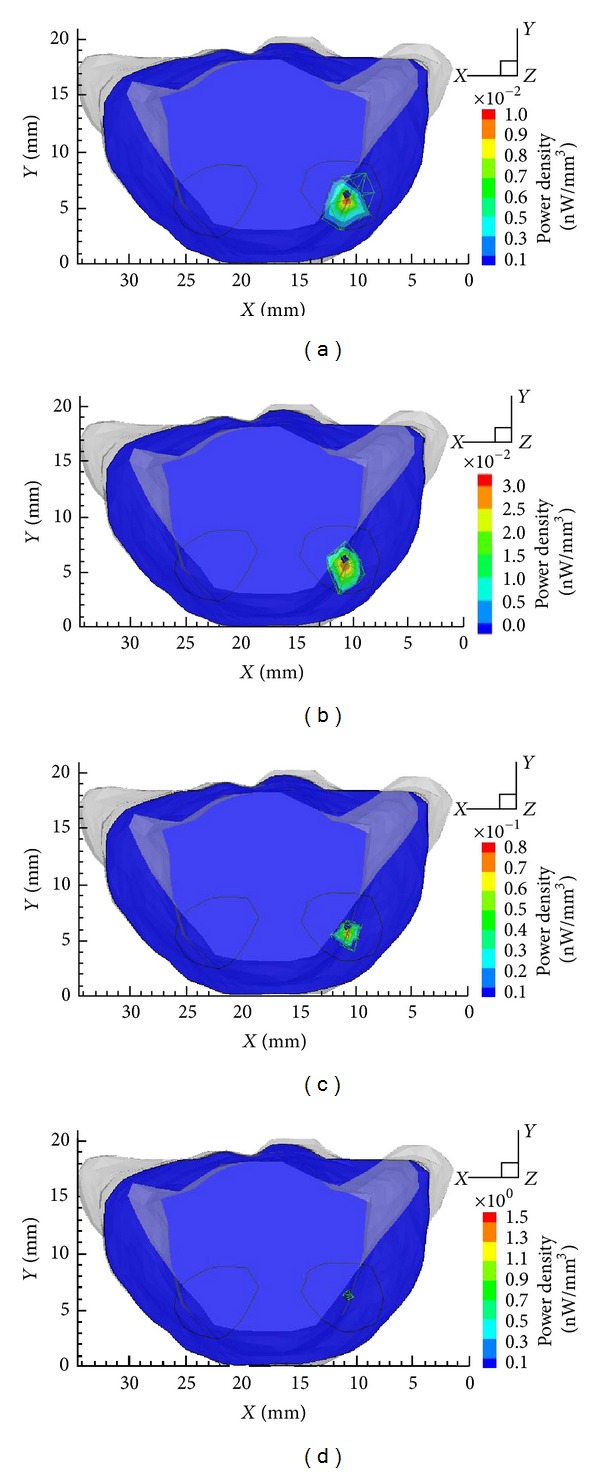
From (a) to (d): transverse views at *z* = 25 mm of the reconstruction results on mesh Θ_1_ to mesh Θ_4_ in single source, respectively.

**Figure 5:**
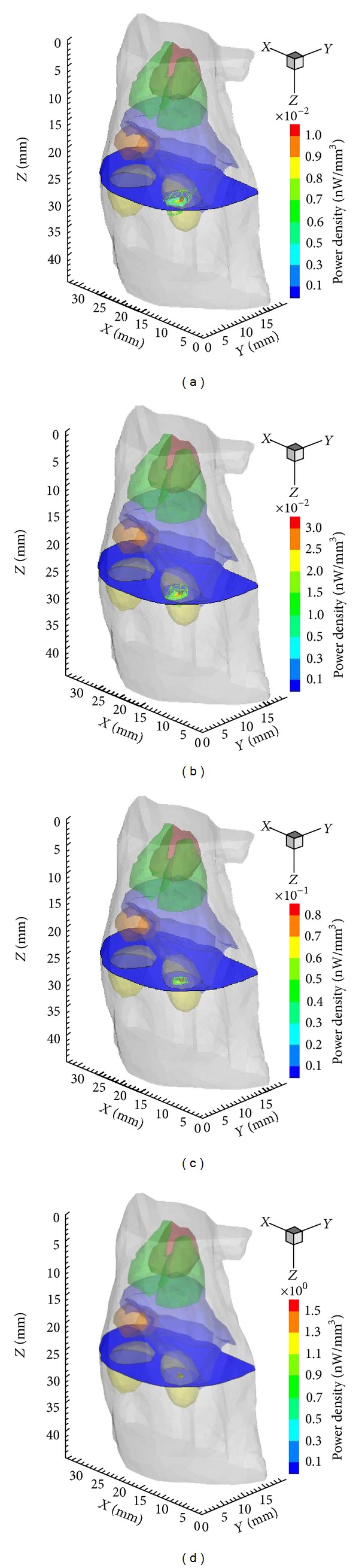
3D views of the reconstruction results on mesh Θ_1_ to mesh Θ_4_ in single source.

Following the proposed hybrid multilevel reconstructions method, the final result in single-source case was obtained by four rounds of reconstructions.  Figure 3 shows the refinement of local mesh around targets and the solution progress from mesh Θ_1_ to mesh Θ_4_. According to the proposed methods, fine resolution only presents around targets, while coarser resolution retains in other regions, which contributes to reaching the desirable resolution at acceptable computational cost. Figures 4 and 5 are the transverse views and 3D views of reconstruction results from mesh Θ_1_ to mesh Θ_4_, which illustrate the improvement of results during adaptive mesh refinement.

To demonstrate the necessity and effectiveness of the hybrid reconstruction scheme, we first compared the SwF-LASSO and IVTCG method on the initial coarse mesh Θ_1_, and then we compared the results of hybrid method, that is, SwF-LASSO + IVTCG, with that of only using SwF-LASSO on the succeeding mesh levels. The detailed reconstruction results are presented in Table 2. Obviously, the reconstruction results by IVTCG are inferior to that of SwF-LASSO on Θ_1_, and hybrid AFEM scheme performs better than the traditional AFEM that uses monoalgorithm of SwF-LASSO on the subsequent mesh Θ_2_ to mesh Θ_4_.

Owing to the hybrid multilevel reconstruction scheme, the location error and the relative error of power distinctly decrease with the adaptively local mesh refinement. Especially, significant improvement of reconstructed density and power can be seen from the results in Table 2 and Figure 4.

### 3.2. Double-Source Reconstruction

**Figure 6:**
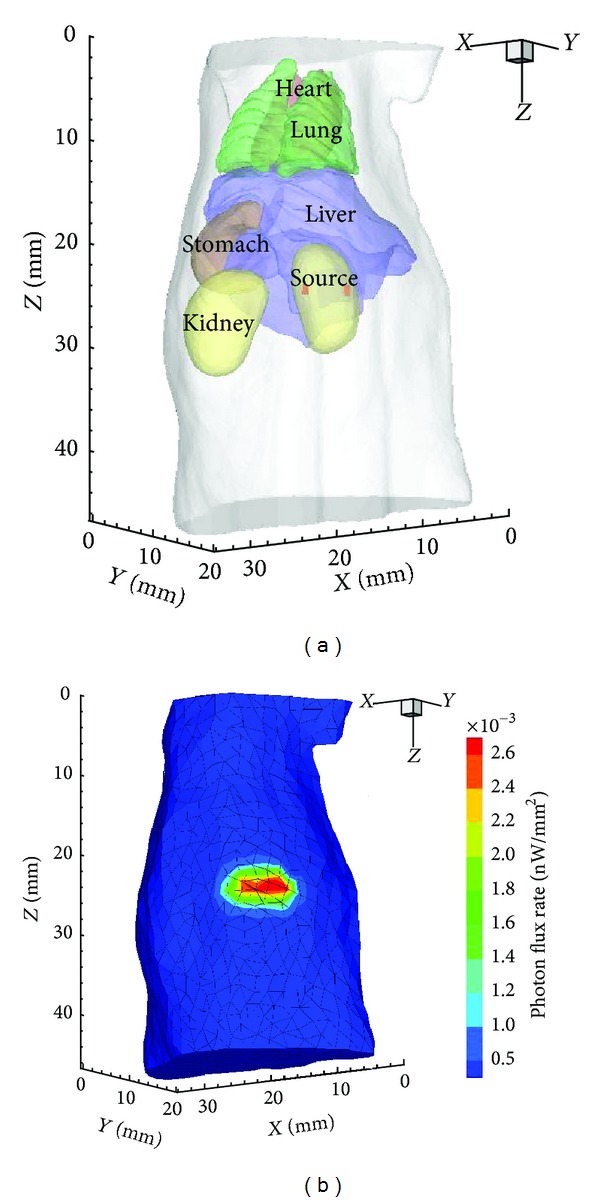
(a) Source setting in double-source case. (b) Initial mesh for reconstruction and the simulated photon distribution on surface.

We also investigated the resolving ability of the proposed method with two closely separated sources. Two cylindrical sources, same as that in the above single-target setting, were located in the right kidney with their centers at (9, 6.5, 25) and (12, 4, 25), respectively. They were identical in size and density, but the initial powers of them were 0.2120 nW and 0.2250 nW, mainly due to the influence of the mesh. The source setting and the simulated photon distribution are shown in Figure 6.

**Figure 7:**
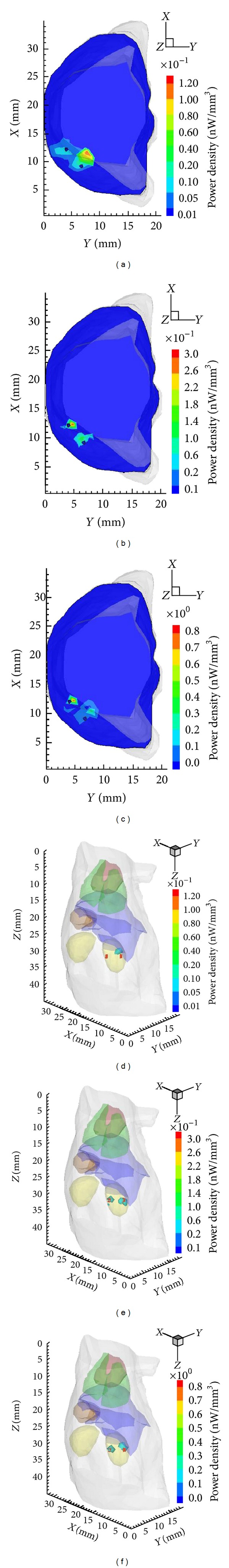
Top row: transverse views of reconstruction results in double-source case at *z* = 25 mm. Bottom row: 3D views. (a) and (d) are the first-round results on mesh Θ_1_, (b)and (e) are the final results of the proposed hybrid method, (c) and (f) are the final reconstruction results by the monoalgorithm of SwF-LASSO.

In double-source case, the multilevel reconstruction terminated on the third mesh level Θ_3_.  Figure 7 displays the reconstruction results by the proposed method on mesh Θ_1_ and mesh Θ_3_. The final result of the traditional AFEM, only using SwF-LASSO as the inversion algorithm on each mesh level, is also shown in Figures 7(c) and 7(f) for comparison. More detailed quantitative results are summarized in Table 3.

Figure 7 witnesses an apparent advantage of using hybrid scheme. Although the first-round result was biased towards a node between the targets on mesh Θ_1_, the proposed method successfully identified the two targets finally, which should be attributed to both the AFEM and the hybrid strategy. By contrasting Figure 7(d) with Figure 7(e), we can observe that the improvement caused by multilevel reconstruction with AFEM is evident. Nevertheless the final result of using monoalgorithm of SwF-LASSO is obviously inferior to that of using hybrid algorithm in terms of location accuracy and the reconstructed power error. Take source 1 for instance, the LE of the hybrid scheme reduces by 0.19 mm and the RE of power falls down to 2.3%. As for source 2, the proposed hybrid reconstruction method yields a 78% plunge in relative error of power.

## 4. *In Vivo* Experiments

To further validate the proposed method, an *in vivo* experiment was performed on an adult nude mouse. The animal procedures were in accordance with the Fourth Military Medical University that approved the animal protocol.

In the *in vivo* experiment, a capillary approximately 1.25 mm in diameter and 4.08 mm in length was inserted into the abdomen of the nude mouse. The capillary filled with 5 *μ*L luminescent liquid served as the testing source in this experiment. The luminescent solution was extracted from a red luminescent light stick (Glow products, Victoria, Canada), and the generated luminescent light had an emission peak wavelength of about 644 nm. The initial total power was 300 nW (the total power = luminescent solution volume × luminescent solution flux density = 5 *μ*L × 60 nW/*μ*L).

**Figure 8:**
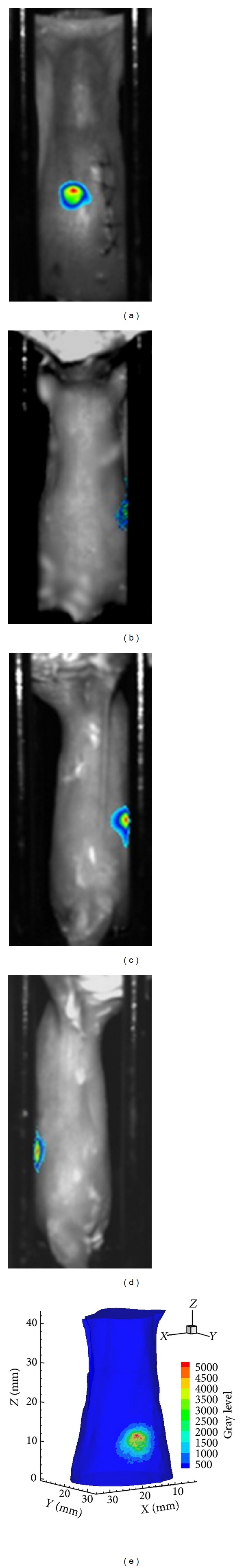
(a)–(d) are multiview superimposed images of photographs and luminescent images, and (e) is the surface mapping result before reconstruction.

This set of BLT experiments were conducted with a dual-modality BLT/micro-CT system [23]. The anesthetized mouse was first photographed, and luminescent images were taken by a calibrated CCD camera from four directions at 90 degree intervals with different exposure times. The multiview superimposed photographs and luminescent images are shown in Figures 8(a)–8(d).

After the optical data were acquired, the intact mouse was scanned using the Micro-CT. Because of the limited field of view, only the torso section was scanned. The volume data were reconstructed using GPU-accelerated FDK algorithm [28]. From the CT slices, we located the center coordinate (21.44, 27.52, 9.76) of the actual luminescent source. The mouse body was segmented into five anatomical components, including muscle, heart, lungs, liver, and kidneys. The relevant optical properties of the mouse are listed in Table 4 [29].

**Figure 9:**
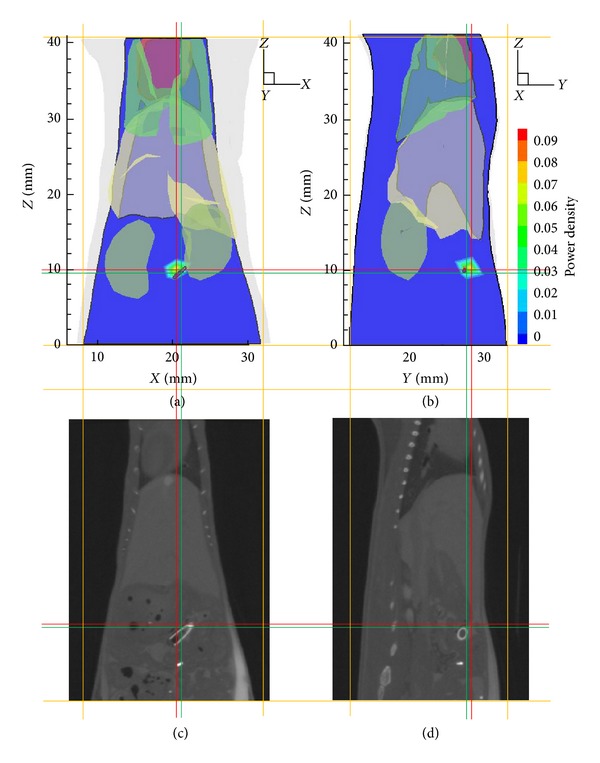
The transverse view of the reconstruction result and the comparison with the corresponding CT slices. The cross of the green lines denotes the actual source center, and the cross of the red lines denotes the reconstructed center.

**Figure 10:**
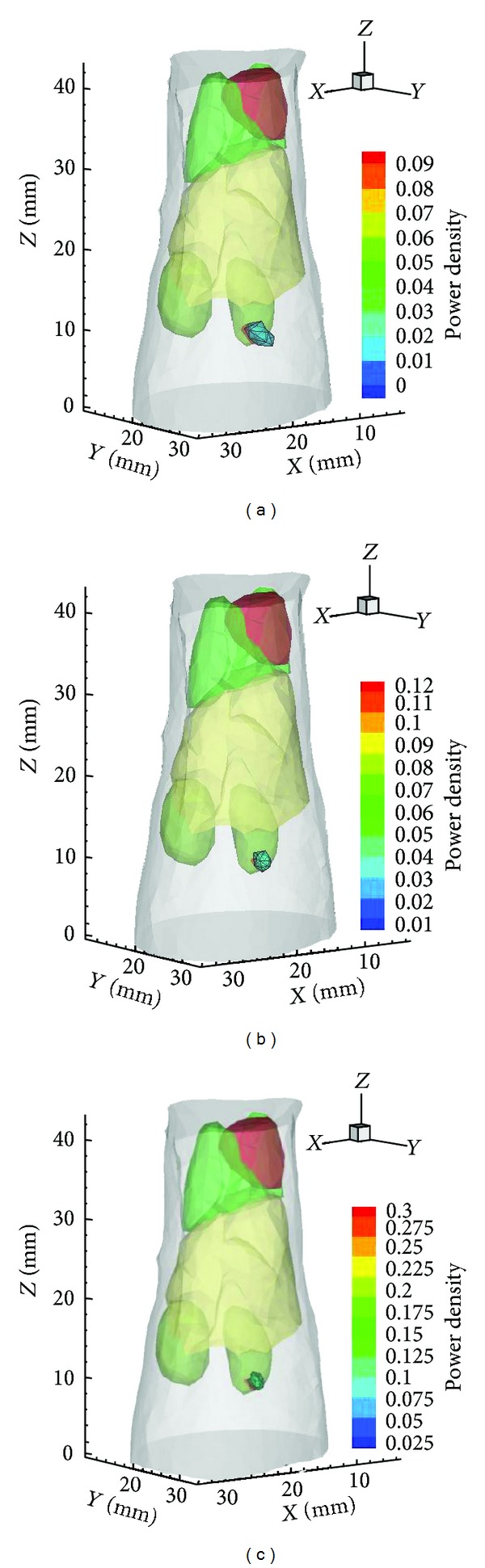
(a)–(c) are the reconstruction results for* in vivo* data on mesh Θ_1_ to mesh Θ_3_.

Based on the collected multiview luminescent images and the volume data of CT, the 3D surface distribution is determined by the mapping algorithm described in [30], as shown in Figure 8(e). After the mapping process, three rounds of reconstructions on gradually refined meshes were performed with the proposed hybrid method. The reconstruction result on mesh Θ_1_ is presented in Figure 9, where the source center is (20.39, 27.98, 9.78) with a deviation of 1.15 mm to the actual center. From mesh Θ_1_ to mesh Θ_3_, the source locations are identical, which means that the SwF-LASSO algorithm yields relatively accurate location from the begging. However, the preliminary reconstruction on the initial coarse mesh Θ_1_ possesses relative bigger errors in source power. After two rounds of local mesh refinement, the final results of hybrid method improved prominently. Specifically, the reconstructed power increased from 149.01 nW to 214.60 nW, and the RE of power decreased from 50.33% to 28.47%. The 3D views of the corresponding results on mesh Θ_1_ to mesh Θ_3_ are presented in Figure 10.

## 5. Discussion and Conclusion

We present a novel multilevel reconstruction method for whole domain BLT, which combines the merit of sparse regularization with the advantage of adaptive FEM. Numerical experiment results employing synthetic data with a digital mouse model illustrate that the proposed hybrid multilevel reconstruction scheme is able to accurately localize and quantify source distribution without *a priori *information of permissible source region and multispectral measurements. The *in vivo* experiments conducted on a nude mouse with a dual-modality BLT/micro-CT system further validate the proposed method.

From the above experiments, we can find that the inversion algorithm on the initial coarse mesh has more important impact on the final result in the proposed hybrid scheme. The SwF-LASSO algorithm is able to provide a good initial localization with better numerical stability, which guides the subsequent reconstruction on finer meshes to obtain more accurate location and power. Furthermore, the experimental results also demonstrate that the hybrid strategy works. Compared with the multilevel reconstruction using monoalgorithm, the hybrid scheme performs better especially for multiple targets reconstruction. Therefore, it is also possible to form another qualified hybrid scheme using some other promising inversion algorithms.

For the sake of computational efficiency, the reconstructions presented in this paper are based on the diffusion equation. Therefore, the inadequately accurate forward model also leads to some inevitable error. The reconstruction performance might be further improved by using more accurate models, which is also the direction of our further work.

In addition to the many advantages of adaptive finite element methods, such as providing fine resolution around targets with coarser resolution in other region, the proposed hybrid scheme has two remarkable features. (i) Reconstruction result evolves adaptively with iterations, and the reconstruction accuracy is easily controlled by users. (ii) The inversion techniques employed on the initial coarse mesh and the succeeding ones vary with the discretization level to maintain solution stability and computational efficiency.

## Figures and Tables

**Table 1:** Optical properties for the atlas organs region.

Material	Muscle	Lungs	Heart	Liver	Kidney	Stomach
*μ*_*a*_ [mm^−1^]	0.23	0.35	0.11	0.45	0.12	0.21
*μ*_*s*_′ [mm^−1^]	1.00	2.30	1.10	2.00	1.20	1.70

**Table 2:** Reconstruction results in single-source case on different mesh levels.

Mesh	Recon. method	Recon. location (mm)	LE (mm)	Power (nW)	RE (%)
Θ_1_	SwF-LASSO	11.02,5.45,25.18	**0.58**	**0.1409**	**38.71**
	IVTCG	10.60,5.38,27.00	2.13	0.0514	77.64
Θ_2_	SwF-LASSO	11.02,5.45,25.18	0.58	0.1711	25.58
	Hybrid method	11.02,5.45,25.18	**0.58**	**0.1773**	**22.88**
Θ_3_	SwF-LASSO	11.02,5.45,25.18	0.58	0.1765	23.23
	Hybrid method	11.02,5.45,25.18	**0.58**	**0.1861**	**19.05**
Θ_4_	SwF-LASSO	11.00,5.99,25.01	0.01	0.1834	20.23
	Hybrid method	11.00,5.99,25.01	**0.01**	**0.2529**	**10.00**

**Table 3:** Reconstruction results on different mesh levels in double-source case.

Mesh	Recon. method	Source ID	Recon. location (mm)	LE (mm)	Power (nW)	RE (%)
Θ_1_	SwF-LASSO	1	10.96,7.63,24.52	2.31	0.5231	147.5
		2	10.96,7.63,24.52	3.81	0.5231	132.5
Θ_2_	Only SwF-LASSO	1	9.98,7.58,25.33	1.50	0.3337	57.4
		2	12.09,4.66,25.06	0.67	0.5805	158
	Hybrid method	1	9.70,6.14,25.10	0.79	0.2208	4.2
		2	12.09,4.66,25.06	0.67	0.2083	7.4
Θ_3_	Only SwF-LASSO	1	9.70,6.14,25.10	0.79	0.2894	36.5
		2	12.09,4.66,25.06	0.67	0.4393	95.2
	Hybrid method	1	8.80,5.94,24.91	**0.60**	**0.2168**	**2.3**
		2	12.09,4.66,25.06	**0.67**	**0.2638**	**17.2**

**Table 4:** Optical properties of the living nude mouse.

Material	Muscle	Lungs	Heart	Liver	Kidney
*μ*_*a*_ [mm^−1^]	0.009	0.460	0.138	0.829	0.155
*μ*_*s*_′ [mm^−1^]	1.258	2.265	1.077	0.736	2.533
